# Greek health professionals’ perceptions of the HPV vaccine, state policy recommendations and their own role with regards to communication of relevant health information

**DOI:** 10.1186/s12889-016-2831-5

**Published:** 2016-06-03

**Authors:** Christina Karamanidou, Kostas Dimopoulos

**Affiliations:** Department of Social and Educational Policy, University of Peloponnese, Damaskinou & Kolokotroni Str, 20100 Corinth, Greece

**Keywords:** HPV vaccine, Health professionals, Policy recommendations, Health information communication

## Abstract

**Background:**

Every year in Europe 60,000 women develop cervical cancer and 30,000 die from the disease. HPV vaccines are currently believed to constitute an important element of cervical cancer control strategy. Currently in Greece, the HPV vaccine is given on demand after prescription by a healthcare professional. Health care professionals’ role is key as they are in a position to discuss HPV vaccination with parents, adolescents and young women. This study is aiming to explore health care professionals’ perceptions of the HPV vaccine, state policy recommendations and their own role with regards to communication of relevant health information.

**Methods:**

This was an in-depth, qualitative study, employing a stratified, purposeful sampling. Fifteen face-to-face, semi-structured interviews were conducted with health care professionals from a variety of disciplines: pediatrics, obstetrics and gynecology, infectious diseases, pharmacy, dermatology, general practice. Thematic qualitative analysis was used to analyze participants’ accounts.

**Results:**

Five major themes were identified: health care professionals’ perceptions towards the HPV vaccine (recognition of importance, concerns about safety, effectiveness and impact of long-term use), animosity between medical specialties (territorial disputes among professional bodies, role advocacy, role limitations), health care professionals’ perceptions of the public’s attitudes (effects of cultural beliefs, health professionals’ attitudes, media and family), the role of the state (health policy issues, lack of guidance, unmet expectations) and their own role (provision of health information, sex education).

**Conclusions:**

Health professionals’ concerns, lack of role definition and uniform information provision have led to territorial disputes among professional bodies and distrust among different medical specialties. Positive and negative judgements deriving from a multitude of sources have resulted in the confusion of the general public, as manifested by low vaccination rates. Due to the lack of clear regulation of vaccination prescription, administration and mode of delivery, factors such as lack of knowledge, cultural beliefs and personal attitudes have shaped the vaccination landscape. These factors have neither been explored nor addressed prior to the initiation of this public health effort and as such there is an evident less than efficient use of resources.

## Background

Every year in Europe 60,000 women develop cervical cancer (cc) and 30,000 die from the disease [1]. Cervical cancer has a younger age of onset compared to other adult cancers and currently ranks as the second most frequent cancer in European women between 15 and 44 years of age [1]. Cytology based screening programs constitute the main tool for detecting precancerous abnormalities and early stages of cancer which if treated can result in a 80 % prevention of cervical cancers [2]. Complementary to secondary prevention, cervical cancer prevention can additionally take place on a primary prevention level, following the recent introduction of the two vaccines against Human Papilloma Virus (HPV), a family of viruses that are extremely common worldwide and are estimated to cause 100 % of cervical cancer cases [2]. HPV vaccines are currently believed to constitute an important element of cervical cancer control strategy and thus a response to a critical public health need [2]. The World Health Organization stresses that ‘ensuring universal access to HPV vaccination, screening and treatment services will be key to reducing the burden of cervical cancer worldwide’ [2].

According to the European Cervical Cancer Association (ECCA) 2009 report, recommendations for HPV vaccination vary across European countries. Certain countries have established or plan to establish school vaccination programs such as the UK, Norway, Sweden, Belarus and Romania while others offer some variation of the ‘on demand’ vaccination plan such as France, Germany, Luxemburg, Portugal, some regions of Spain and Greece. In Greece, the recommendations for HPV vaccination were put together in 2008 by the Ministry of Health and Social Solidarity after appropriate suggestions from the National Vaccination Committee [3]. Currently, the HPV vaccine may be prescribed to girls aged 12 to 18 years by physicians in the specialty of Pediatrics, Obstetrics-Gynecology, General Practice and Pathology. For ages 18 to 26 years, the vaccine may be prescribed by medical specialties of Obstetrics - Gynecology, General Practice, Pathology and Dermatology or Venereology [4]. Currently there are two vaccines available: GARDASIL, a quadrivalent vaccine against HPV types 16, 18, 6, 11 and CERVARIX, against HPV types 16 and 18.

Preliminary evaluations show that school-based vaccination programmes tend to achieve higher coverage compared to on demand vaccination provision, even if this is supported by health professionals’ direct invitation or public education programmes [3]. Statistical data reveal 92 % coverage in Scotland and 90 % in Spanish regions where HPV vaccination is delivered through a school program while on demand vaccination supported by invitation and public education efforts puts coverage at approximately 50 % in the Netherlands and lower than 50 % in Spanish regions where provision on demand is not supported by educational activities [3]. In summary, countries which offer HPV vaccination on demand tend to achieve suboptimal coverage rates with lower socioeconomic groups and minorities being at a disadvantage, thus perpetuating health inequalities [3].

In Greece, recently conducted research studies have revealed that the incidence of HPV infection can range from 22.7 %, as found in a sample of 225 women attending an outpatient gynecology clinic [5] up to 50.7 %, as found in a large epidemiological study in a sample of 2952 women [6]. However, HPV vaccination coverage has been shown to be very low among female students 25.8 % from various disciplines (*n* = 3.153) and relatively low 44.3 % even among female higher education students (*n* = 865) in medicine, nursing and allied health professional study programs [7], who as aspiring health professionals are in an advantageous position with regards to comprehending the need for the adoption of a health innovation [8]. Furthermore, the vaccination coverage percentage is even lower among Greek adolescents, namely 11.9 % [9].

With regards to the factors affecting Greek women’s intention to get vaccinated or to vaccinate their children, research shows that receiving information from a health care professional positively affected intention to self-vaccinate [10] while factors such as: younger age, higher educational level, previous visits to the gynecologist, consistent condom use, easy access to healthcare, non-smoking status and single relationship status, constituted positive predictors of vaccination [8]. As far as the reasons for declining vaccination are concerned, women have listed ‘fear of vaccine safety’ as the main reason and moreover were less likely to vaccinate if they had received information from the mass media compared to other sources (e.g. Hellenic Center for Disease Control and Prevention) [7]. In contrast, women’s main reason for declining vaccination was initially ‘insufficient knowledge’, however this was later substituted by ‘fear of vaccination adverse events’ [10]. Interestingly, this change occurred after the introduction of the vaccine, and coincided with negative media publicity [10].

The role health care professionals’ in communicating health information concerning the HPV vaccine and in shaping parental, adolescent and young women’s attitudes towards the said vaccination has been highlighted in the international literature [11–15]. For the majority of the general public, health care providers still constitute a formal source of information regarding any health issue while they are also an important advocacy group with scientific status and presence in the media [16].

The acceptance of the vaccines is of paramount importance when it comes to the successful implementation of HPV vaccination programs. Both adolescents who are the primary target group and their parents who are the decision makers need to see the vaccine in a positive light so that uptake but also adherence to all three doses is ensured [11]. Specifically, findings of a Danish study [12] revealed that young women (16–26) listed recommendation by a health care professional among the reasons for HPV vaccination along with prevention of cervical cancer, parental encouragement and being close to someone with cancer. According to a review of the US literature [13] parents were more likely to opt for vaccination after receiving a recommendation from a health care professional which influenced young adolescent girls’ vaccination decision. Furthermore, interactions between health professionals and parents play a major role in forming parental attitudes towards vaccination [14]. Finally, findings of a French study [15] suggest that health professionals have a significant role to play in information provision as they are in a unique position to discuss with the target population barriers to acceptance.

The exploration of health care professionals’ perceptions is particularly salient in countries where HPV vaccines are provided on demand after prescription. To the best of our knowledge, there have been no research studies to date investigating perceptions of health care professionals in Greece since the release of the HPV vaccine in 2008. Specifically, the current study is aiming to explore the following research questions:

1. What are health care professionals’ perceptions towards the HPV vaccine?
2. What are health care professionals’ perceptions towards state policy recommendations?
3. What are health care professionals’ perceptions of their own role with regards to health information communication?

## Methods

### Design

This was an in-depth, qualitative study. Fifteen face-to-face, semi-structured interviews were conducted with health care professionals. A stratified, purposeful sampling was employed to include participants from a variety of disciplines relating to HPV infection, HPV vaccination and cervical cancer. Patton [17] describes stratified samples as samples within samples and suggests that purposeful samples can be stratified or nested by selecting particular units or cases that vary according to a key dimension. In this case the sample was stratified in that participants varied according to discipline/ specialty. A maximum of three participants were selected from each relevant discipline, resulting to a total sample of 15. The sample was also purposeful as sample participants were recruited through key institutions which the investigators’ thought ought to be present in the sample namely the two largest medical schools in the country University of Athens & University of Thessaloniki, the National School of Public Health and the Hellenic Center for Diseases Control and Prevention (KELPNOO).

### Materials

The interview schedule consisted of 26 open-ended questions which covered topics such as the health professionals’ perceptions of the HPV vaccine (e.g. effectiveness, safety), state policy recommendations (e.g. mode of delivery), health information sources (state, professionals, media), modes of health information communication, vaccination decision making, sexual health education, adolescent sexual behavior, perceptions of public’s and health professionals’ beliefs. The interview schedule was developed after identification of key issues of interest through a thorough literature search as well as attendance of relevant educational meetings and conferences (i.e. meeting of the Greek HPV society in Thessaloniki, 2012).

### Participants

Health care professionals came from a range of specialties such as: pediatrics, obstetrics and gynecology, infectious diseases, pharmacy, dermatology and general practice. Five participants were female and ten were male. Their age ranged from 34 to 65 years. All participants worked full time and the majority additionally owned their own private practice. Pharmacists were an exception as they all owned community pharmacies, a very common occurrence in Greece.

### Procedure

Participants were recruited from a variety of different contexts, namely hospitals, universities and organizations and were invited to participate over the phone. None of the invited participants approached refused to participate. An appointment for the interview was arranged after the nature and aim of the study was explained and informed consent was obtained. The interviews took place in a private office in each participant’s workplace, lasted approximately 30 to 40 min and were conducted by the principal investigator (CK), who is a health psychologist. The interviews were conducted in the Greek language, were audio recorded and transcribed verbatim. For confidentiality purposes, participant names were replaced by an abbreviated version of their discipline and their age (Please see list of abbreviations at the end of the manuscript). The Greek General Secretariat for Science and Technology (GSRT), the public body which funded the current study, allocated its evaluation to independent international reviewers/experts through a system of blind reviewing. These international experts among other criteria, reviewed the study aim, design and materials according to the principles included in the manual entitled ‘Ethics for Researchers’, of the European Commission, issued for the 7th Framework Programme (FP7), and approved it without raising any ethical and/or other considerations. Furthermore, following review of the study the University of Peloponnese, School of Social and Political Sciences Research Ethics Committee confirmed that it did not require ethics approval and was in full accordance with the current Research Ethics guidelines.

### Analysis

The audio-recordings were transcribed in Greek and analyzed in the Greek language by both investigators separately. Findings were then compared and an agreement on the final themes was reached. Specific quotes and theme titles were subsequently translated in English by the principal investigator (CK). A separate translation was conducted by an Applied Linguist with an MA qualification. Disagreements on terminology and choice of wording were negotiated and resolved. This method i.e. translation by two separate translators has been suggested by cross-cultural researchers such as Esposito [18]. Furthermore, according to recommendations by van Nes et al. [19] in order to reduce the loss of meaning and thereby enhance the validity of cross-English qualitative research, researchers should stay in the original language ‘*as long and as much as possible’*. The reason for this is that thinking and language are closely related and thus analysis may be influenced if conducted in a language different other the researchers’ own. Finally, according to a review on social research translation issues [20] among the factors affecting the quality of translation in those cases, where the researcher and the translator are the same person, are: the autobiography of the researcher-translator; the researcher’s knowledge of the language and the culture of the people under study and the researcher’s fluency in the language of the write-up. In the present case, both investigators are Greek, hold PhD degrees, were educated in UK and have experience in conducting qualitative research. The investigators were reflective about the design, conduct and analysis of this study. A number of strategies were employed to enhance credibility of the research findings such as ‘*acknowledging biases in sampling, using investigator triangulation, seeking out similarities and differences across accounts to ensure different perspectives are represented and including rich and thick verbatim descriptions of participants’ accounts to support findings’* [21].

Thematic qualitative analysis [22] was used to analyze participants’ accounts. After familiarization with the data, initial codes were generated. Themes were identified, reviewed and defined. Finally, these were analyzed and supported with extracts from participants’ accounts.

## Results

Some of the most prominent themes that emerged from participants’ accounts were the following:

### Health professionals’ perceptions of the HPV vaccine

The majority of participants considered the HPV vaccine ‘*an amazing innovation*’ (PED, 65), described it as ‘…*one of the most important vaccines of the last ten, fifteen years*… (ID, 53) and prescribed it to their patients ‘*I think it is one of the most effective vaccines which have brought change in the area of infections….change in the sense that a very common cancer can be prevented… very few vaccines are related to cancer prevention. Based on this, I consider it’s really important and I recommend it’* (ID, 47).

However, some participants felt there were still issues to consider such as, in one participant’s words: ‘*It is a relatively new vaccine, which means that we can’t be 100 % certain about what it does, its effectiveness or whether it has any adverse effects…*’ (ID, 40). Additionally, some participants had specific concerns pertaining to the HPV vaccine and the long-term effects of its wider use: ‘*Yes, it is a new vaccine…. studies that have been conducted are retrospective, not prospective…so who knows what will happen after twenty years…the technology of production… with a modified protein..I don’t know what kind of effects it might have in auto-immune type illnesses …the virus has too many types…the vaccine only two, the other four…I wonder with natural selection fifteen, twenty years after mass vaccination, if other types will prevail as it has happened with other vaccines*’ (PED, 55). These concerns naturally affected health professionals’ behavior and in some cases gave rise to conflicting advice towards the public: ‘*We administer it but we do not recommend it*’ (PH, 34).

### Animosity among medical specialties

Another strong theme that emerged from the data was the evident animosity among medical specialties. Since the introduction of the HPV vaccine, professional bodies representing health care professionals have been competing for territory, as illustrated by the following quote: ‘*There has of course been a huge battle and from the point of view of the professional association of dermatologists, so that we are also able to prescribe it…and essentially, we would very much like to keep it to ourselves, this vaccine, because…it is us that deal with warts’* (DERM, 48).

Furthermore, participants from different specialties claimed expertise and advocated their own role in dealing with HPV and the HPV vaccine. The distrust between gynecologists and pediatricians was especially prominent. Specifically, pediatricians argued they are the only ones regularly dealing with vaccines as well as receiving and treating patients of the appropriate age groups: ‘*Vaccinations are not in the area of gynecologists and obstetricians. And so they say ‘leave it and we will see…let us have some more years of experience and we will see’. They don’t do much reading. I am really sorry to say this but pediatricians are the most informed doctors. They are well read, (they know) all the results of the multi-center studies that have been conducted…they are alert…to convince parents that they should do it* (PED, 65) compared to gynecologists: ‘*Those of course, that administered it profusely, without even knowing what it is, are gynecologists! Who have no clue about vaccines!’* (PED, 55). Furthermore, gynecologists argued that they are more qualified as they are in a position to diagnose and treat cervical cancer: ‘*It certainly falls into the gynecologist’s area!....The pediatrician is more…addicted to vaccinations*!’ (GYN, 65).

In certain cases, participants realized the limitations of their own role: ‘*Vaccines, although one could say that, as a subject, it is an infectious disease subject, that’s where they belong, but, in order to be applied, they should be given to primary care doctors who come in contact with the public*’ (ID, 40). Conversely, pharmacists and general practitioners who are routinely asked for their advice by members of the public, within the context of a long-term and trusted relationship, expressed the desire for an enhanced role so that they can respond in a responsible manner.

### Health professionals’ perceptions of the public’s attitudes

Participants mentioned a number of different causal factors in an attempt to interpret the low levels of vaccination coverage in Greece. The majority thought the public’s negative attitude is not unique to the HPV vaccine: ‘*I believe that, in Greece, there is generally… public ignorance and a hesitancy towards vaccinations. There is a belief that vaccinations are commercial products of multi-nationals and are designed to bring profit to certain companies and that vaccinations are harmful and anyhow…And sometimes it is difficult to “sell” a vaccine to a patient because vaccinations are meant to be seen from the point of view of community protection rather than individual patient protection’* (ID,40). To substantiate this claim, participants stated that the Greek public also treated the swine flu vaccinations with suspiciousness in 2010. Despite recommendations by KELPNOO (Hellenic Center for Diseases Control and Prevention) and several appeals made to the public, voluntary turnout was very low. Some participants speculated that this negative attitude towards vaccinations might be rooted in the Greek culture and hence encountered repeatedly: ‘*I believe in two factors, in the innate fear of the Greek population of everything new – as far as vaccinations are concerned-…, and also insufficient information….both from the media, if you like, but also from us doctors…to this population*’ (ID,53).

Other participants thought that the public’s attitudes might be affected by health professionals’ attitudes towards the HPV vaccine: ‘*I believe that this reaction by parents, is supported by the refusal of many health professionals.*’ (ID,47) or might be mirroring health professionals’ attitudes: *‘…the public’s distrust we observe … reflects the doctors’ distrust*!’ (PED,65). Participants thought that this alone can justify low vaccination rates: ‘*Doctors are not convinced yet, I guess, otherwise they would (prescribe)…this is up to the doctor’* (PH,58). According to participants this was also the case for H1N1 vaccinations: ‘*Η1Ν1, this flu caused a lot of damage. Because the public thought that this was a profit making game, that Greece obtained a lot of vaccines, towards which there was distrust…from the medical world, as very few hospital doctors and nursing staff actually got vaccinated. So when the medical world, does not accept something…how will (the public)?…not many children were vaccinated… because doctors were not convinced for the need for mass vaccination*’ (PED, 65).

Participants also thought that the public suffered from a lack of reliable information or even misinformation mainly due to the fact that the source is traditional media and the internet. Although they distinguished between traditional media and the internet in terms of credibility: ‘*I have not seen inaccuracies in the press…In the internet…things are out of control*!’ (GYN, 50), they judged this to be problematic because the views expressed via the media can be either positive or negative and could very well shape the public’s reserved attitude towards vaccinations and enhance their resistance to health professionals’ recommendations: ‘*If someone googles it…he/she will see thousands of things against the vaccine. It will certainly affect… (public’s attitudes)…because nowadays people perform thorough searches on the internet. But they cannot be selective about the information that is offered to them. So if they read it is has been connected with, I don’t know, autism, neurological diseases, they will think ‘why perform a vaccination when my child does not have sexual intercourse?*’ (ID, 47).

Participants also highlighted that the Greek family is quite conservative and overprotective which has a number of implications. Specifically, parents are unwilling to openly discuss sexual safety because they refuse to accept the fact that their daughter might be having sexual relations. Therefore, this issue is not addressed early enough: ‘*You essentially miss the critical time period… A mother must be very aware and progressive to consider the fact that her 14-year old daughter will have her sexual debut soon, so that she arranges a visit to the gynecologist*’ (ID,47).

Health professionals were aware that the target population namely teenagers are unique in the sense that they are not very knowledgeable or careful about health issues: ‘*Youth is combined with carelessness about health issues…you are invulnerable, yes. And this is absolutely normal. A normal mentality’* (ID, 47). However, they thought that being vaccinated against HPV will not make teenagers prone to riskier sexual behavior. This is mainly due to the fact that teenagers may not be aware of HPV since it is a relatively new ‘scare’ while other STDs and specifically HIV have already shaped behavior such as condom use: ‘*I believe that children who do not take precautions think about STDs such as syphilis, herpes, vaginal infections…and everything else transmitted sexually. I don’t think that all children know, when they are about to have sexual intercourse, about the HPV virus. This is the last concern. In general they are afraid of AIDS, the other sexual, infectious STDs, unfortunately there are incidents. So, it is unlikely that they preoccupy themselves with cancer since AIDS dominates’* (PED, 65).

### Health professionals’ perceptions of the role of the state

Participants raised a number of issues with regards to the current health on the HPV vaccination policy adopted by the Greek state. Firstly, that the lack of financial resources has shaped this policy to a large extent (i.e. vaccine offered only to girls, no school vaccination program in place, lack of walk-in clinics for STD testing etc). Furthermore, that this policy allows for a serious ‘gap’ in vaccination as health services attendance of girls between 14–25 years of age is minimal due to the fact that these girls have stopped visiting their pediatrician but have not yet paid their first visit to the gynecologist. In addition, they reported that they all suffered from lack of guidance from the Ministry of Health and the state with regards to prescription, administration and mode of delivery (i.e. medical specialties entitled to prescribe the vaccine, recommended age range, conditions of prescription e.g. before or before and after sexual debut, before or after a HPV DNA test, insurance funds covering the cost etc.). As illustrated by one participant: ‘*The ministry in Greece, unfortunately for all issues, sends circulars, which are communicated in print to the responsible structures but in the end never reach the practitioner’* (ID, 40).

Further to this, participants had expectations that they would be supplied with uniform information, materials and resources as: ‘*It goes without saying that the ministry is the one that will direct efforts*’ (DERM, 48). Further to health professionals’ lack of relevant information, participants believed that the public also suffered from lack of information since none recalled a nationwide state initiative or state run campaign for cervical cancer prevention (i.e. screening, vaccination) or a sexual health education campaign targeting teenagers. Finally, the role of the state, as seen by participants, is to actively coordinate efforts in order to achieve large scale immunity: ‘*Since the ministry and the department of public health, have a bigger financial capacity than any private or smaller organization…to regulate something, launch or promote something…a huge social organization, which is independent and formal*’ (GYN, 43).

### Health professionals’ perceptions of their own role

The majority of participants were of the opinion that not only is it within the physicians’ role but rather it is their duty to inform members of the target group about the HPV vaccination and subsequently administer it, irrespective of any personal concerns they might have. As one participant put it: ‘*I believe that, medicine in this day and age is not a science where everybody can do what they want. It is a science! And someone who is practicing medicine ought to respect the rules and the medical stance of this time. And the stance now is to administer the vaccine*’ (ID, 40). Some participants would be willing to extend their role in HPV vaccination or even accept a new role, such as pharmacists who can currently only administer the vaccine upon receipt of a physician’s prescription: ‘*We wish that we pharmacists had this type of responsibilities, to do such a thing (inform)…but as things are today…the system…it is the doctor who is entitled’* (PH, 34).

However, not all participants agreed on whether basic sex education is within the physician’s role. Some thought it best to provide at least some context to the HPV vaccination: ‘*I consider it to be part of my job. Because my job is not tertiary…at tertiary level, in an intensive care unit. My job involves mainly prevention. In my primary care office’* (GYN, 50), while others thought that this was the responsibility of the parent or the state: ‘*These are things that…the doctor cannot do. It is not the doctor’s job to do such a thing. It is not mine either…will I conduct sexual education for my patients? I will do it for my child, not my patient!* (DERM, 48).

Participants’ views converged in that sexual education in Greece is either non-existent or badly conducted partly due to the fact that no-one is clear about whose responsibility it is (i.e. school, family or health professional). All participants agreed that sexual health information should definitely be offered as part of a continuous school sexual health program so that that every child receives the same information via a trustworthy source such as a school physician or nurse, an institution currently abolished: ‘*There is no medical service at schools…in the past there was a medical service, there were doctors, responsible for talking to kids…and the parents, they were invited, but now I don’t know who will be burdened with this engagement, this additional engagement. And there is some resentment, in the medical world due to severe cuts that our salaries have suffered….* (PED, 65), rather than being left to non-expert individual initiatives: ‘*I had an experience, whereby a biology teacher said….(to the schoolchildren) “you don’t need to have it, because you will damage your health…find a person who will only be with you and you will only come in contact with him all your life so you will be fully covered”*’ (GYN, 50).

## Discussion

Findings of the present study indicated that participants recognized the HPV vaccine as a major health innovation. However, they had certain concerns with regards to its safety, effectiveness and impact of long term use. A recent study investigating physicians’ intentions to vaccinate girls at the recommended age range, found that these intentions were largely influenced by subjective norms (i.e. recommendations given to them as appropriate clinical practice) [23]. Specifically, that physicians tended to agree with recommendations of esteemed colleagues, professional organizations etc. The authors concluded that when new practices such as technological health innovations are introduced it is imperative for physicians to receive guidance and recommendations by their professional bodies and/ or the state. These findings are especially relevant to countries that have not introduced school based vaccination programs but rather rely on health professionals’ recommendations for vaccination, such as Greece. Further to this, current findings revealed that the HPV vaccine has given rise to a great amount of animosity among health professionals from various medical specialties. The lack of inter-professional respect within health care settings is a well documented phenomenon which regrettably is likely to increase the public’s distress and can compromise its safety [24].

A number of studies have suggested that health professionals need to be supported in their role of initiating a discussion about HPV, offering vaccination and arriving at a shared decision with their patients [25, 26]. A large part of this process would involve eliciting perceptions and addressing concerns with the help of good informational resources [14]. Health professionals in the present study felt the need to inform their patients in a responsible manner, however lacking in guidance, information and resources. This finding is in line with that of a previous study [27] investigating the experiences of UK school nurses during the first year of the HPV school vaccination program. School nurses reported that, at the start of the program in 2008, they felt unprepared as they had not received enough training beforehand and attributed this to lack of planning on the part of the policy makers.

Certain interventions aiming to support health professionals have been proposed in the literature. Specifically, one Irish study suggested the use of an intervention within the context of which a range of factors found to influence HPV related clinical behaviors are addressed. Such factors include beliefs about consequences, knowledge, emotion and social influences [25]. Another Canadian study investigated the use of decision boxes namely tools offering guidance to clinicians with regards to communicating evidence-based information to patients, exploring patients’ values and preferences in management options to support a shared decision-making process regarding ‘medical questions that have no single best answer’ [26]. Prototypes of these decision making tools, available both in print and online, were evaluated by both physicians and patients via focus group discussions and modified accordingly to include issues of interest such as benefit and harm of an intervention, list of questions to ask the health professional etc. HPV vaccination was among the clinical topics suggested by health professionals as suitable for this approach.

Some health professionals in the current study believed that talking about sexual health to adolescents in the context of a visit was outside their medical role. In contrast, findings of a similar previous study suggested that pediatricians, general practice physicians and family practice physicians were willing to discuss sexual health issues with their young patients using HPV vaccination as an opportunity [28]. However, once again the importance of supporting physicians in order to conduct these conversations is highly stressed. The authors suggest that support can take the form of skills training to help physicians build rapport, elicit information regarding the patient’s behavior, give information in age-appropriate ways, assess the relevance and importance of said topics to individual patients etc.

Finally, none of the participants of the present study could recall a state run health promotion campaign with regards to HPV and cervical cancer. According to WHO, public knowledge about cervical cancer and its association with HPV can be improved via the use of community education campaigns, a solid strategy for increasing vaccine acceptance [2]. Furthermore, it is highlighted that the information/ education messages should be tailored to the local cultural context and needs of the target population (i.e. adolescents and young adults) but also to the needs of parents or guardians, educators, community leaders and health-care providers. A recent US study evaluated a social marketing campaign aiming to raise awareness among parents with regards to HPV vaccination [29]. The campaign which addressed perceptions of susceptibility to HPV infection, severity of cervical cancer, benefits of vaccination as well as barriers to vaccination such as safety, efficacy, cost and access was planned, implemented and evaluated by communication and public health experts. Within the context of the campaign, HPV vaccination material was placed in medical settings for providers to use when discussing this issue with patients. The majority of providers used the campaign brochures and thought the campaign influenced parents’ decisions to get their daughters vaccinated against HPV. The campaign was deemed successful after the monitoring of the campaign’s website, hotline calls, public services announcements, cross-sectional surveys and immunization rates [29].

This study’s findings need to be interpreted with caution as purposeful sampling was employed. The sample was not representative of Greek health professionals as a whole, but rather was selected to include participants from a range of relevant medical specialties and institutions. Findings have highlighted some important issues in current practice, which could be investigated further in future qualitative and quantitative research. Moreover, findings have led to the formulation of practice and policy recommendations.

The successful introduction and acceptance of the HPV vaccines will depend on a range of factors including public knowledge, attitudes towards the vaccine and health information communication. The prevention, screening and treatment of the HPV virus and cervical cancer constitute an area requiring the input of multiple disciplines both in terms of research and practice. Firstly, the state needs to make available a policy document, outlining the rationale of evidence-based recommendations leading towards a clear public health goal. Furthermore, multi-disciplinary expert groups need to be established, comprising of all disciplines relating to the issue of HPV vaccination, in order to develop guidelines and protocols for health care professionals with regards to informing patients, screening for eligibility, suggesting vaccination, prescribing, vaccinating, performing follow- ups etc. It has been suggested in the literature that in some cases professionals’ own knowledge will not be sufficient to cover patients’ informational needs and deal with their concerns [30]. Therefore, health professionals need to be supported through various educational initiatives which will help them communicate health information effectively while respectfully considering patients’ perspectives. Furthermore, health professionals also need to be given access to the latest medical research developments, as well as provided with support tools (e.g. a structured approach to communicating vaccination information according to patients’ readiness to vaccinate) [14], shared decision making tools (e.g. decision boxes for medical issues with no single best answer) [26] etc.

Currently underutilized resources need to be identified and exploited such as community pharmacists, who frequently constitute the first port of call for health related inquiries. Their frequent contact with the public can allow them to take on new roles such as infection prevention [31]. Moreover, the institution of school nurses and doctors needs to be re-introduced, a change which will further reinforce the school’s role as a health promotion setting. With their input, sexual health education can be taught and sexual promotion programs can be carried out on a permanent basis. According to the CDC, nurses can play a major role in preventive health care and to this end has produced educational material for them to use to educate parents and talk to adolescents [32]. In conclusion, the introduction of the HPV vaccine as a public health effort needs to be closely monitored and further research needs to be conducted to assess its effectiveness and impact.

## Conclusions

This study constitutes a mapping of Greek health professionals’ perceptions of the HPV vaccine, state policy recommendations and their own role. One of the major emerging issues is the fact that there are no established, effective communication channels among the various professional bodies, between state institutions and health professionals or among the different relevant medical specialties. Another major issue identified is that the state policy as it currently stands lacks clarity. With no school vaccination program in place, health professionals have been assigned the responsibility to inform, prescribe and administer the HPV vaccine to the target population. However, the state has not provided guidance, information or resources. The impact of this lack of effort coordination is twofold: firstly, health professionals’ concerns, lack of role definition and uniform information provision have led to territorial disputes among professional bodies and distrust among different medical specialties. Secondly, positive and negative judgments deriving from a multitude of sources have resulted in the confusion of the general public, as manifested by low vaccination coverage rates. Due to the lack of clear regulation of vaccination prescription, administration and mode of delivery, factors such as lack of knowledge, cultural beliefs and personal attitudes have shaped the vaccination landscape. These factors have neither been explored nor addressed prior to the initiation of this public health effort and as such there is an evident less than efficient use of resources. Some of the issues described in this paper could be relevant to other European countries which have also adopted ‘on demand’ HPV vaccination schemes or are currently considering an HPV vaccination policy.

## Abbreviations

CC: cervical cancer
CDC: Center for Diseases Control
DERM: dermatologist
ECCA: European Cervical Cancer Association
GSRT: General Secretariat for Science and Technology
GYN: gynecologist
HPV: human pappiloma virus
ID: infectious disease specialist
PATH: pathologist
PED: pediatrician
PH: pharmacist
WHO: World Health Organization
